# A compact single channel interferometer to study vortex beam propagation through scattering layers

**DOI:** 10.1038/s41598-019-56795-z

**Published:** 2020-01-15

**Authors:** Sruthy J. Lathika, Vijayakumar Anand, Shanti Bhattacharya

**Affiliations:** 1 Department of Electrical Engineering, Indian Institute of Technology Madras, Chennai, 600036 India; 2 Present Address: Centre for Micro-Photonics, Faculty of Science, Engineering and Technology, Swinburne University of Technology, Hawthorn VIC, 3122 Australia

**Keywords:** Engineering, Optics and photonics

## Abstract

We propose and demonstrate a single channel interferometer that can be used to study how vortex beams propagate through a scatterer. The interferometer consists of a multifunctional diffractive optical element (MDOE) synthesized by the spatial random multiplexing of a Fresnel zone plate and a spiral Fresnel zone plate with different focal lengths. The MDOE generates two co-propagating beams, such that only the beam carrying orbital angular momentum is modulated by an annular stack of thin scatterers located at the focal plane of the Fresnel zone plate, while the other beam passes through the centre of the annulus without any modulation. The interference pattern is recorded at the focal plane of the spiral Fresnel zone plate. The scattering of vortex beams through stacks consisting of different number of thin scatterers was studied using the proposed optical setup. Conflicting results have been reported earlier on whether higher or lower charge beams suffer more deterioration. The proposed interferometer provides a relatively simple and compact means of experimentally studying propagation of vortex beams through scattering medium.

## Introduction

Light propagation through scattering and turbid medium produce speckle signatures from which it is often difficult to extract useful information. Different techniques have been developed in order to look through both static as well as dynamic scatterers^1–5^. These techniques can be broadly classified into two types. In the first type, optical signal processing^6–8^, correlation techniques^9–13^ and phase retrieval algorithms^14,15^ have been developed to decode a speckle signature and convert it into useful information. A second direction of research is to develop adaptive aberration correction methods to compensate for the disturbance introduced by the scattering medium^16,17^. In the latter approach, structured light beams with scatter-resistant characteristics are used to reduce the aberrations introduced by the scatterer^18,19^.

Vortex or orbital angular momentum (OAM) beams have a unique complex amplitude, which makes them attractive for various applications. For example, Laguerre-Gaussian (LG) beams have been used in optical trapping^20^ and optical communication^21^ experiments. Besides, LG beams have also shown scattering-resistant properties in various studies^22–25^ and several techniques have been developed to aid probing and communicating through scattering medium using LG beams^26,27^. In most of these techniques^25,28^, the deterioration of the phase of the beam is studied using an interferometer. With the need for interferometry, the footprint of the experiment increases, as more components and vibration isolation, have to be added to the experiment. There are a few interference-less optical configurations and techniques used to measure the level of deterioration of the LG beam^24,26^ but the information is incomplete without measurement of phase.

In this manuscript, we propose a single channel optical configuration based on a multifunctional diffractive optical element (MDOE) for studying the robustness of LG beams of different charges versus level of scattering. The advanced optical configuration does not require a vibration isolation system, beam splitting, etc., and therefore, is extremely compact. Secondly, the interferogram is designed to directly show whether the charge of the beam changed during propagation, in terms of the number of radial intensity lobes. In that sense, the measurement of the deterioration of LG beams through thin scatterers can be considered a pattern recognition problem. The single channel interferometer has been implemented to study and compare the scattering characteristics of LG beams with different topological charges when interacting with single or multiple thin scattering layers.

Several groups have reported theoretical and simulation studies of OAM beam propagation in a scattering medium. While the results depend on several factors including the type of scattering media under study, the characteristics of the OAM beam propagated, and the performance parameters measured; more detailed studies are required to resolve the many different results presented so far. The compact interferometer is proposed as an elegant means by which to carry out further studies in this important field.

## Methods

**Figure 1:**
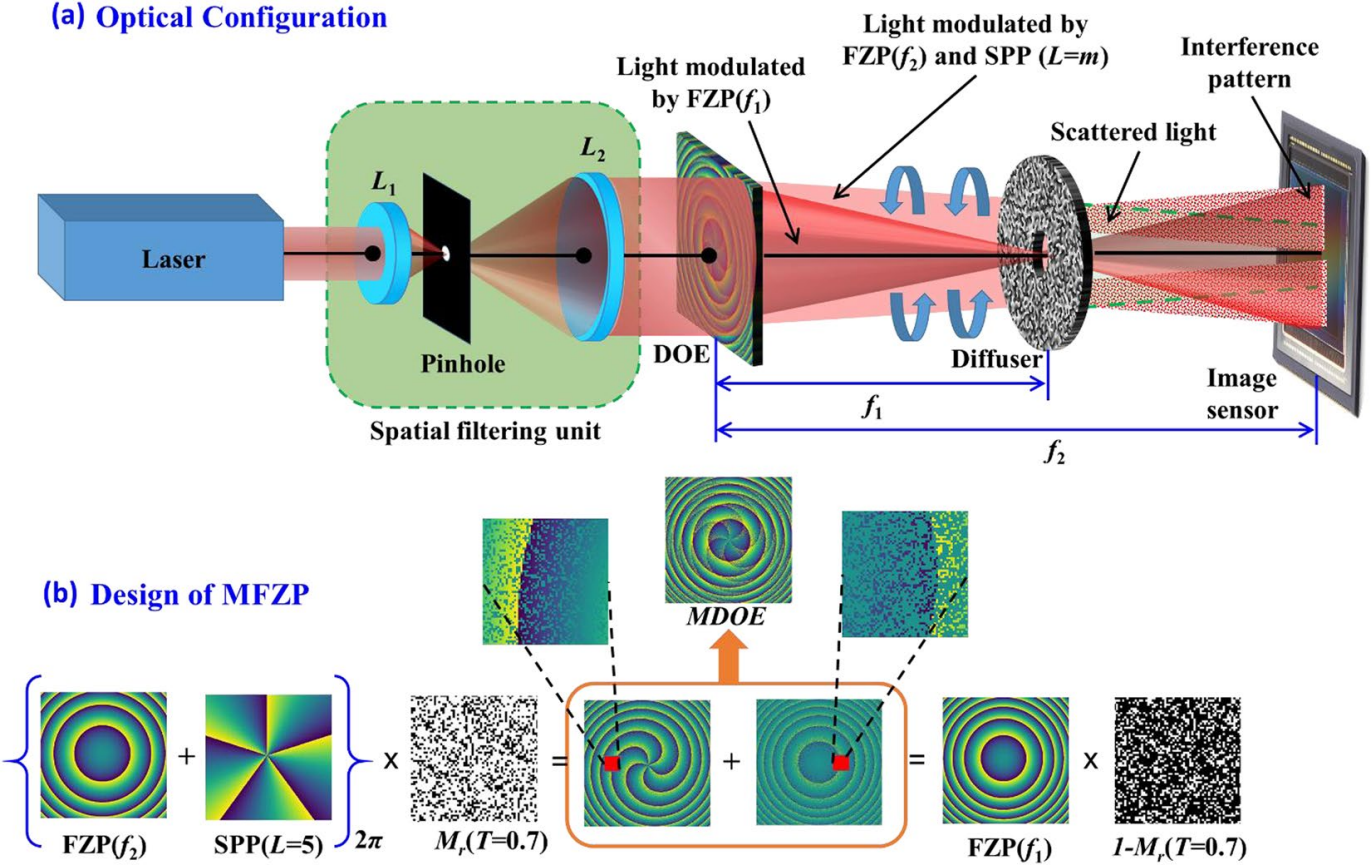
(**a**) Optical configuration of the single channel, dual beam interferometer. The light from the laser is spatially filtered and collimated by a pinhole and refractive lenses *L*_1_ and *L*_2_. (**b**) Technique by which the MDOE is designed.

The optical configuration of the single channel, dual beam interferometer is shown in Fig. 1(a) and the synthesis of the MDOE, with phase ${\phi }_{MDOE}(x,y)$, is shown in Fig. 1(b). The MDOE is synthesized by random multiplexing of two diffractive functions, namely a Fresnel zone plate^29^ and a spiral Fresnel zone plate^30–32^ with different focal lengths. The phase of the Fresnel zone plate (*FZP*) is given by ${\Phi }_{FZP}(f)=\{\,-\,{\rm{\pi }}/{\rm{\lambda }}f\}{R}^{2}$, where *R* is the radial coordinate and the focal length *f* = *f*_1_. On the other hand, the phase of the spiral Fresnel zone plate is given by ${\Phi }_{SFZP}(f,L)={[{\Phi }_{FZP}(f)+L\theta ]}_{2{\rm{\pi }}}$, where *L* is the topological charge, *f* = *f*_2_ and *θ* is the azimuthal angle in the beam’s cross section. The focal length values are selected such that *f*_1_ < *f*_2_ and the scatterer is positioned at the plane corresponding to *f*_1_, as shown in Fig. 1(a). The two functions are multiplexed in the manner shown in Fig. 1(b). The resulting phase distributions at each step of the process are also shown there. *M*_*r*_(*T*) represents a virtual mask comprising a binary random pattern [0,1] synthesized with a transmittivity *T*, where *T* is given by *N*_1_/(*N*_1_ + *N*_2_). *N*_1_ and *N*_2_ are the number of pixels with value 1 and 0 respectively. As the virtual mask is applied directly to the spiral phase through the operation $\exp [j{\Phi }_{SFZP}]{M}_{r}(T)$, *T* can also be considered to control the fraction of the incident energy directed into the LG beam. A larger value of *N*_1_, would increase the intensity of the LG beam with respect to the reference beam. Therefore, we will henceforth refer to *T*, as the splitting ratio.

Collimated light from a coherent source is incident on the MDOE. This generates two co-propagating beams with complex amplitudes $exp[j{\Phi }_{FZP}]\{1-{M}_{r}(T)\}$ and $\exp [j{\Phi }_{SFZP}]{M}_{r}(T)$. At the focal plane *f*_1_, the former beam is focused to a spot, whereas the latter occupies a larger annular region. There is therefore, no overlap between the beams. On the other hand, at the focal plane *f*_2_, the two beams spatially overlap resulting in an interference pattern, with intensity given by

1 $${I}_{1}={|{{\rm{E}}}_{1}+{E}_{2}|}^{2},$$

where *E*_1_ is a diverging spherical wavefront,

2 $${E}_{1}=\Im \{[1-{M}_{r}(T)]\exp [j{\Phi }_{FZP}]\exp [j\frac{\pi {R}^{2}}{\lambda {f}_{2}}]\}\,$$

In Eq. (2), *R* is the radial distance from the axis. Neglecting complex constants results in the following expression for the spirally varying phase front *E*_2_,

3 $${E}_{2}=\Im \{{M}_{r}(T)exp[-j{\Phi }_{SFZP}]\}.$$

A deeper look at the formation of the interference pattern reveals many interesting characteristics of the compact interferometer configuration. In any two-beam interference-based phase measurement technique, the interferogram is processed and the phase is retrieved using phase retrieval algorithms. In the proposed technique, the two wavefronts that interfere in the sensor plane are a diverging spherical wavefront and a helical wavefront. The diverging spherical wave has a constant phase for one value of radius, as the azimuthal coordinate is varied from 0 to 2π. On the other hand, the helical wavefront has a linear phase variation along that same path. Therefore, the resulting interference pattern is a direct representation of the relative phase difference between the two waves. Consequently, a maximum value in the interference pattern represents a phase difference of 0 and a minimum value in the interference pattern represents a phase difference of π. The number of peaks along any ring of infinitesimal thickness represents the topological charge of the helical wavefront.

In order to study the effect of scattering, a stack of annular scatterers, with phase ${\Phi }_{S}(x,y)$ in an annular region, is introduced with the center of the stack lying on the optical axis at focal plane *f*_1_. The index *p* is used to denote the number of layers in the stack. The resultant scattering phase therefore, is the modulo-2π phase addition of *p* scattering layers (that form the stack). The intensity at the plane *f*_2_ will be given by ${I}_{2}={|{{\rm{E}}}_{1}+{E^{\prime} }_{2}|}^{2}$, where the complex amplitude of the scattered vortex beam is

4 $${E^{\prime} }_{2}=\Im \{exp[j{\phi }_{s}]\Im \{{M}_{r}(T)exp[-j{\Phi }_{SFZP}]\exp [j\frac{\pi {R}^{2}}{\lambda {f}_{1}}]\}\exp [j\frac{\pi {R}^{2}}{\lambda ({f}_{2}-{f}_{1})}]\}.$$

The degree of deterioration of the vortex beam can be measured by a cross-correlation between *I*_1_ and *I*_2_ in comparison to the autocorrelation of *I*_1_. It is well-known that autocorrelation of a function gives the narrowest peak while the deviation from this ideal function can be quantified against the degree of deterioration. From the previous explanations, cross-correlation measures the degree of deterioration of the phase profile with respect to the ideal one and therefore is a direct and simpler method compared to the phase retrieval methods.

## Experiments

### Fabrication of the MDOEs

**Figure 2:**
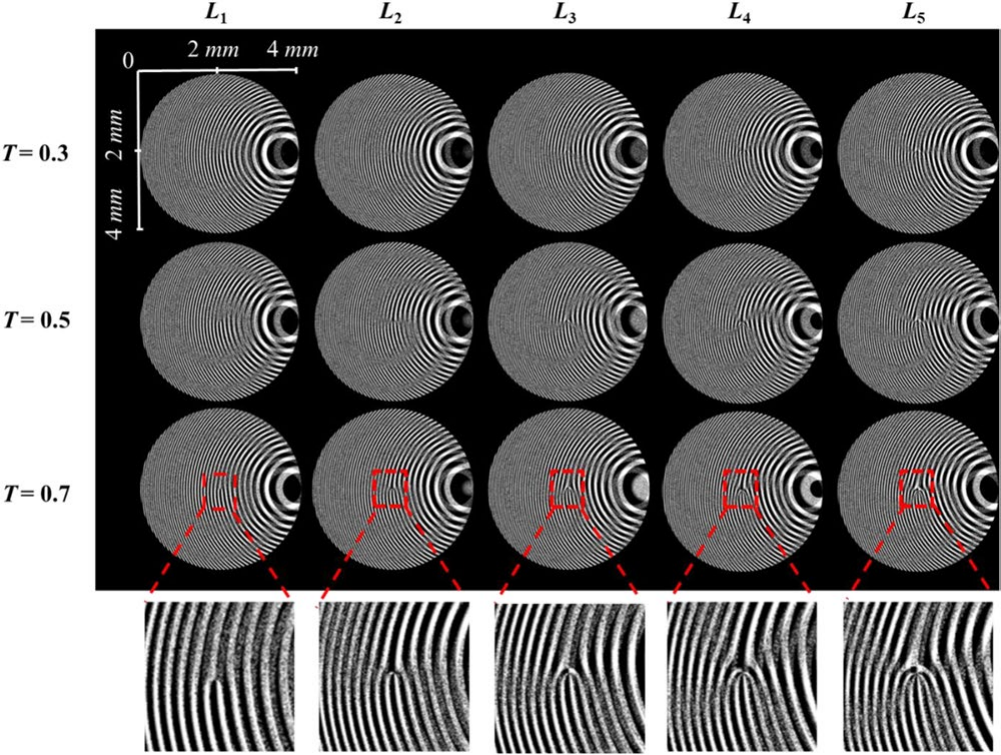
Images of MDOEs for different topological charges *L* = 1 to 5 and splitting ratios *T* = 0.3, 0.5 and 0.7 designed with two-phase levels and a linear phase of *α* = 0.03 radians. The number of lines in the fork pattern increases with the topological charges.

MDOEs were designed as binary elements and therefore, the calculated function ${\Phi }_{MDOE}$ is binarized to have only two phase values [0, π]^32^. Since, binarization produces a zeroth diffraction order when there is a phase error during fabrication, an additional linear phase with an angle *α* = 0.03 radians was added to the MDOEs such that the required optical signal is moved away from the optical axis. The images of the design files for MDOEs with *T* = 0.3, 0.5 and 0.7 for topological charges *L* = 1 to 5 are shown in Fig. 2. The central area is magnified to clearly show the increase in the number of forks with a corresponding increase in the topological charge *L*. When *T* = 0.5, the incident energy is divided equally between both the diffractive functions, whereas for other *T* values, one function of the MDOE will dominate changing the splitting ratio. The fabrication procedure is presented in Supplementary Section S1.

### Experiments

**Figure 3:**
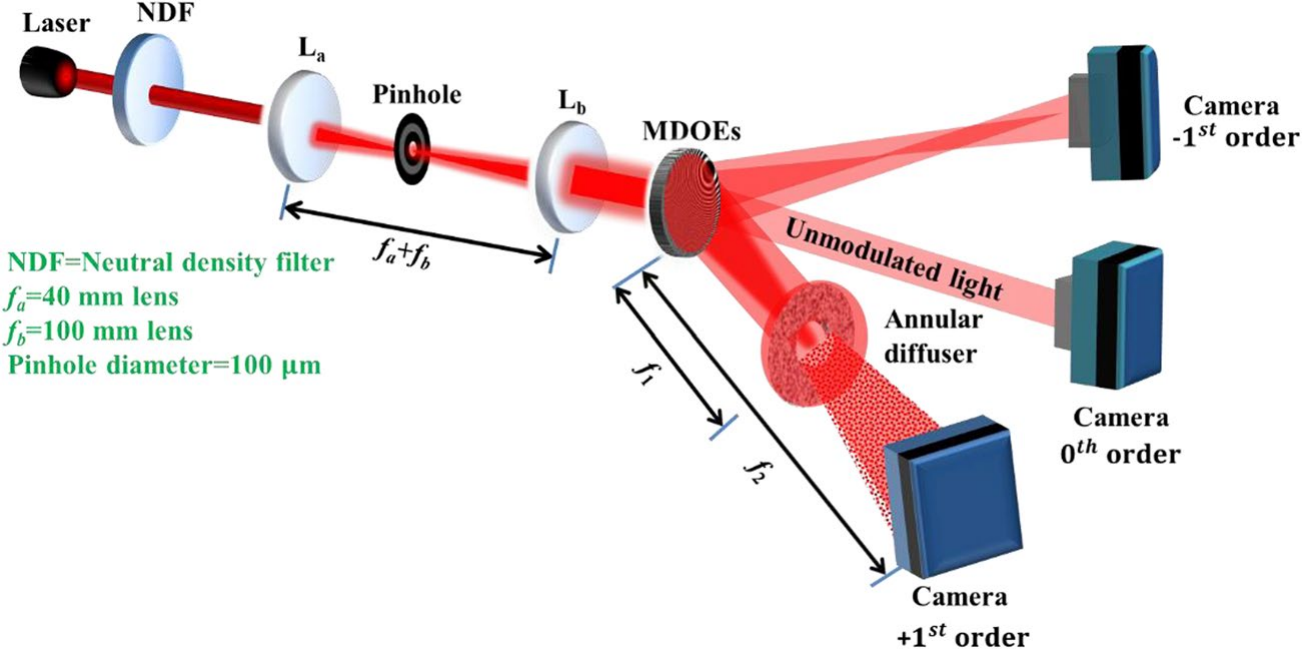
Experimental setup for the evaluation of scatterers using the MDOE as a single channel interferometer.

**Figure 4:**
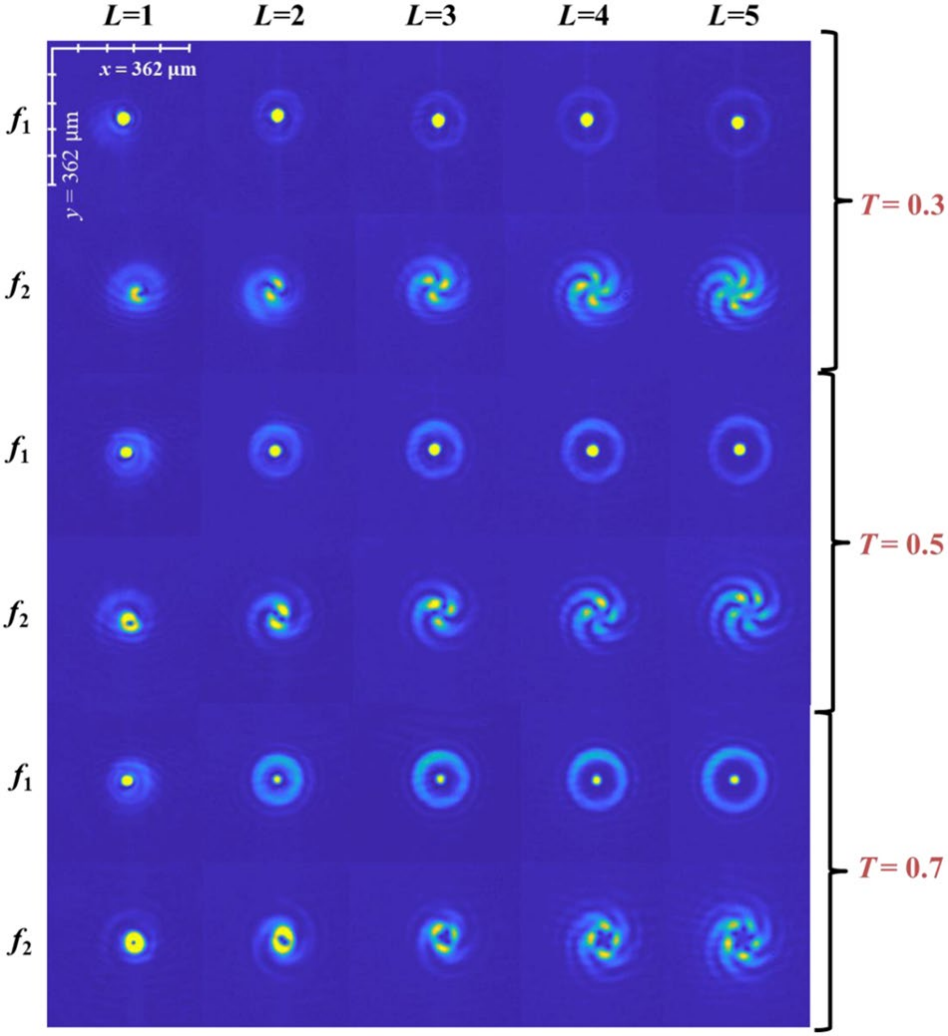
Recorded intensity patterns without the scattering layers for different topological charges *L* = 1 to 5, splitting ratios *T* = 0.3, 0.5 and 0.7 and at two planes namely *f*_1_ and *f*_2_.

The experimental set up is shown in Fig. 3. Light from a He-Ne laser (*λ* = 632.8 *nm*) is spatially filtered using a pinhole with a diameter of 100 *µm* and a refractive biconvex lens *L*_*a*_ (*f*_*a*_ = 40 *mm*). The spatially filtered light is collimated by a second refractive biconvex lens *L*_*b*_ (*f*_*b*_ = 100 *mm*). The collimated light is incident on the MDOE, which generates several orders. Of interest are the spherical converging beam and a spiral-spherical beam, with focal lengths 25 *cm* and 30 *cm* respectively, generated in the +1^st^ order. An image sensor (Thorlabs camera with 1024 × 768 pixels of size 4.65 *µm*) mounted at a distance of 30 *cm* from the MDOE captures the interference of these two beams at this plane. The interference pattern will be between a spherical reference beam and either a scattered and or an unscattered LG beam, depending on whether or not the annular scatterer stack is introduced at a distance of *f*_1_ (25 *cm*) from the MDOE. A study of the scattering characteristics of the scatterer stack is presented in Supplementary Section S1. A neutral density filter (NDF) was used to reduce the light intensity and record the interference patterns without saturating the image sensor. The recorded intensity patterns in the two planes in the absence of a scatterer for different topological charges and splitting ratios are shown in Fig. 4.

**Figure 5:**
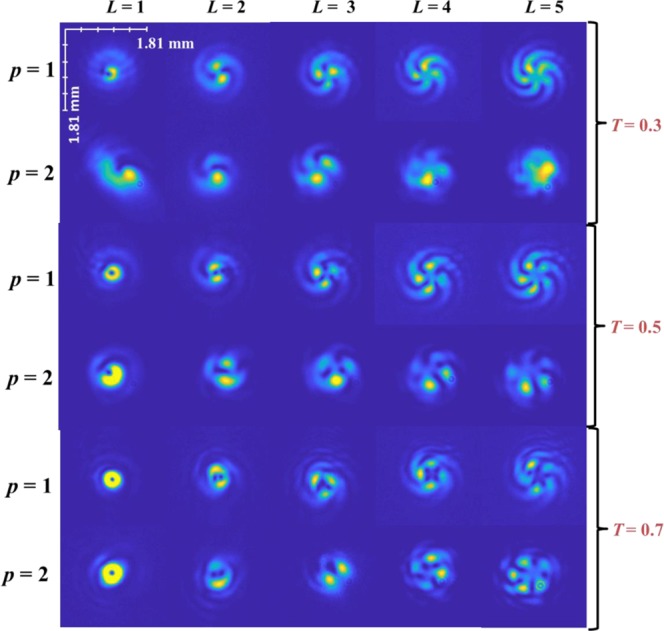
Recorded intensity patterns at plane *f*_2_ with the scatterer at *f*_1_. The first rows present the results for one (*p* = 1) and two (*p* = 2) scatterers, whereas the columns are the results for the topological charges *L* = 1 to 5. The experimental results for different splitting ratios (*T* = 0.3, 0.5 and 0.7) are presented in subsequent rows.

Following this, the stack of scatterers is introduced in the plane *f*_1_ and the interference pattern is once again recorded at the plane *f*_2_. The images of the recorded interference patterns for the different topological charges and splitting ratios are shown in the Fig. 5. Two sets of interference patterns are displayed in this figure, those taken with one or two scattering layers, corresponding to *p* = 1 and *p* = 2 respectively.

The first point to note is that the resulting interference pattern contains a number of intensity lobes *m* along the azimuthal direction that match the topological charge *L*. In the experiment, an LG beam of known charge is sent through the scatterer; any change in the charge of the beam would be easily picked from the number of lobes in the interference pattern.

From Fig. 4, it is seen that when the splitting ratio *T* is increased, the relative intensity of the two beams in the plane *f*_1_ varied resulting in the ring pattern around the central spot becoming brighter. This is not surprising, as *T* controls the amplitude of the LG beam, as described earlier. Figure 5 presents the interference patterns at plane *f*_2_, with the scatterer in place. It is clear that the disturbance to the LG beam (that is the beam that sees the scatterer) causes the interference pattern to deviate from the case without the scatterer. The deviation is even more pronounced for the case two scattering layers (*p* = 2). It is also observed that the deterioration to the interferogram is greater, when the LG beam’s amplitude is weaker (*T* = 0.3). The experimental results were verified by simulations, presented in Supplementary Section – 2.

**Figure 6:**
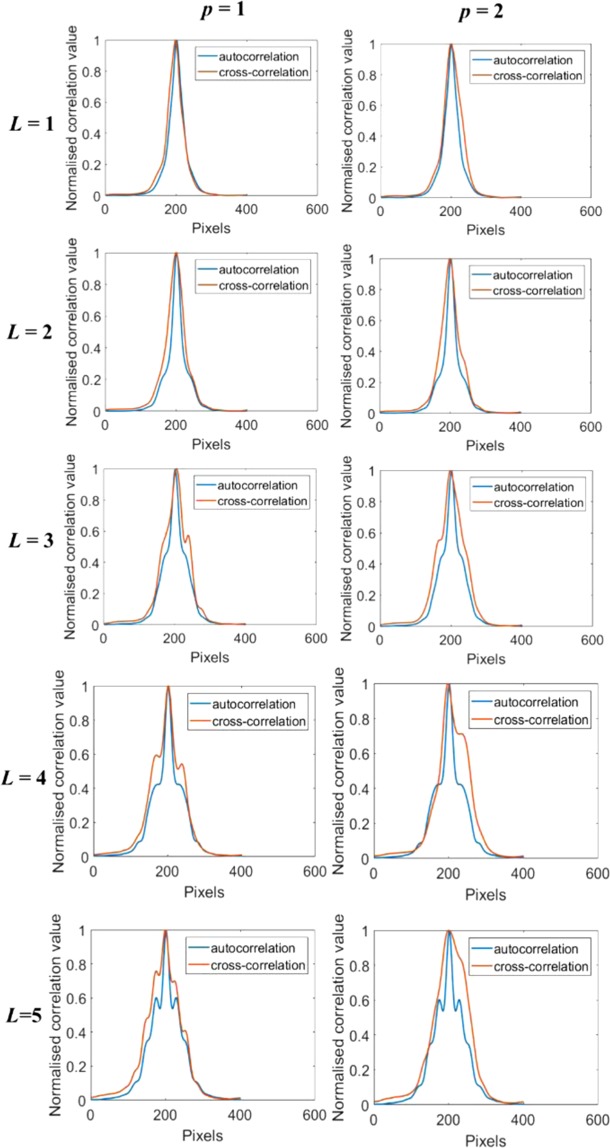
Plot of the cross-correlation results for the scattering layers (*p* = 1, *p* = 2) for different topological charges *L* = 1 to 5 and splitting ratios *M*_*r*_ = 0.5.

**Figure 7:**
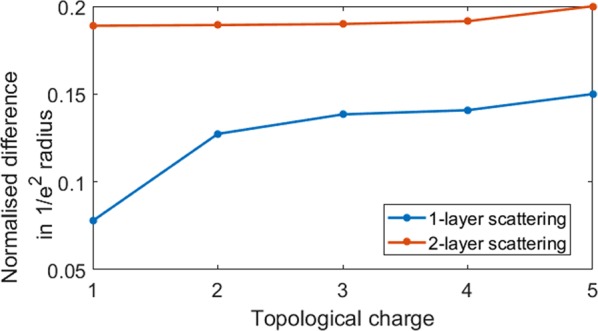
Plot of the difference between the 1/*e*^2^ radius of the cross-correlation and autocorrelation plots for topological charges *L* = 1 to 5 normalized with the corresponding autocorrelation radius, for the scattering layers (*p* = 1, *p* = 2) splitting ratio *T* = 0.5.

The conventional method for the study of scattering characteristics using phase retrieval algorithm is given in Supplementary Sections 4 and 5. In order to quantitatively compare the degree of deviation of the scattered case from the un-scattered one, the interference pattern recorded with the scatterer is cross-correlated with the interference pattern recorded without the scatterer. Cross-correlation function measures the similarity between two patterns. When two patterns are identical a maximum value is obtained (autocorrelation) and vice versa. If the vortex beam is not distorted then the interference pattern remains the same resulting in a sharp correlation function and when it is distorted, the correlation function becomes broad. To have a reliable study, the cross-correlation function is compared with the autocorrelation function without scatterer^33^. They are compared with the corresponding autocorrelation results of the un-scattered OAM beam. The cross-correlation results for *p* = 1 and *p* = 2 for *T* = 0.5 are shown in Fig. 6. The difference between the 1/*e*^2^ radius of the cross-correlation and autocorrelation plots for topological charges *L* = 1 to 5 are calculated and normalized with the corresponding autocorrelation radius. This is plotted in Fig. 7. The deviation of the cross-correlation function from the autocorrelation increases with the number of scattering layers. It is also found that for *p* = 1 case, as the topological charge increases, the cross-correlation result has more deviation from its corresponding auto-correlation result. This says that lower charge OAM modes are less deteriorated by the scattering layer. It would be because we have used a dense scatterer, which is expected to deteriorate the stability of the propagating OAM mode strongly, resulting in fracturing of the OAM mode^34,35^. However, in the *p* = 2 case, the range of topological charge under study seems to be affected in the same way, which is why the deviation is more or less a constant with a slightly increasing trend.

## Summary and Conclusions

We have proposed and demonstrated a compact, single channel interferometer using a MDOE for the study of propagation of beams with orbital angular momentum through scattering layers. The MDOE generates two complex waves, where OAM is carried by only one of the waves and the other wave is used as a reference. The MDOE generated a focal plane suitable for modulating only the vortex, while transferring the reference wave without any modulation. The optical configuration was designed such that in an axial plane, the topological charge can be measured from the number of intensity peaks. In most existing optical configurations^25,28^, the study of beam propagation through scatterers involves bulky optical configurations with a minimum of two optical channels and many optical components. In the proposed method, the entire interferometer consists of only one optical element. The proposed single channel interferometer with a MDOE along with a blind cross-correlation method was found to measure the degree of deterioration of the beams carrying OAM. The experimental results match with the results expected with the scatterers used. Clearly, the results would change dramatically according to the type of scattering media being studied. This conclusion is supported by the simulation results presented in the supplementary material. In particular, Supplementary Fig. 8 shows an improvement in the quality of the interference pattern (between reference and the scattered OAM beam) with an increase in the scattering ratio, for constant phase retardation. However, Supplementary Fig. 9 indicates exactly the opposite behavior, as the phase retardation was increased along with the scattering ratio. A previous study^36^ indicated that the scattering degree of OAM beams with higher topological charges was higher when they were incident on diffuser, which confirms the preliminary study carried out by the compact interferometer. More thorough studies and comparative analysis of different charged beams in scattering medium are needed. We believe that the proposed set-up and MDOE will allow exactly this. The efficiency of the demonstrated MDOE is limited due to the binary random multiplexing method and the efficiency can be increased by fabricating a greyscale version of the elements.

## Supplementary information

Supplementary Information2
