# Supplementary material for: A compact single channel interferometer to study vortex beam propagation through scattering layers
Source: Sci Rep. 2020 Jan 15;10:296. doi: 10.1038/s41598-019-56795-z (PMC6962366; doi:10.1038/s41598-019-56795-z)
Supplement: Supplementary file 1 — Supplementary Information2 [file 41598_2019_56795_MOESM1_ESM.pdf]

A compact single channel interferometer to study vortex beam propagation through scattering layers

J. L. Sruthy,[1] A.Vijayakumar[2] and S. Bhattacharya[1]

[1]Department of Electrical Engineering, Indian Institute of Technology Madras, Chennai 600036, India.
[2]Centre for Micro-Photonics, Faculty of Science, Engineering and Technology, Swinburne University of Technology, Hawthorn VIC 3122, Australia.

S. 1 Fabrication of MDOEs

The design patterns were transferred to chromium coated mask plates using laser fabrication method in a conventional mask writer. The images of the mask patterns are shown in supplementary figure S1. The amplitude masks were used for the fabrication of two level binary phase elements using UV lithography on borofloat glass (t_g=0.5 *mm*) coated with SU8-2002 NPR (MicroChem) with an index of refraction 1.58 for λ=632.8 *nm*. The resist layer was coated with a spin speed of 5000 *rpm*, acceleration of 500 *rpm*/*s* and time of 30 *s* followed by a prebaking at 95°C for 1 *min*. The UV exposure was about 60 mJ/cm^2 followed by a post exposure baking at 95°C for 2 *min*. The resist was developed by MicroChem's SU-8 developer for about 1 *min* and rinsed in IsoPropyl Alcohol and dried in nitrogen gas. The fabricated devices were hard baked at 120°C for 30 *min*. The fabrication process is shown in figure S2. The microscope images of the fabricated elements are shown in the figure S3. The darker regions seen in the figure S1 and figure S3 are not due to underdevelopment of the resist but due to the spatial random pattern, which scatters light. A magnified version of the central area in the figure S3 shows that the dark regions are not actually underdeveloped.

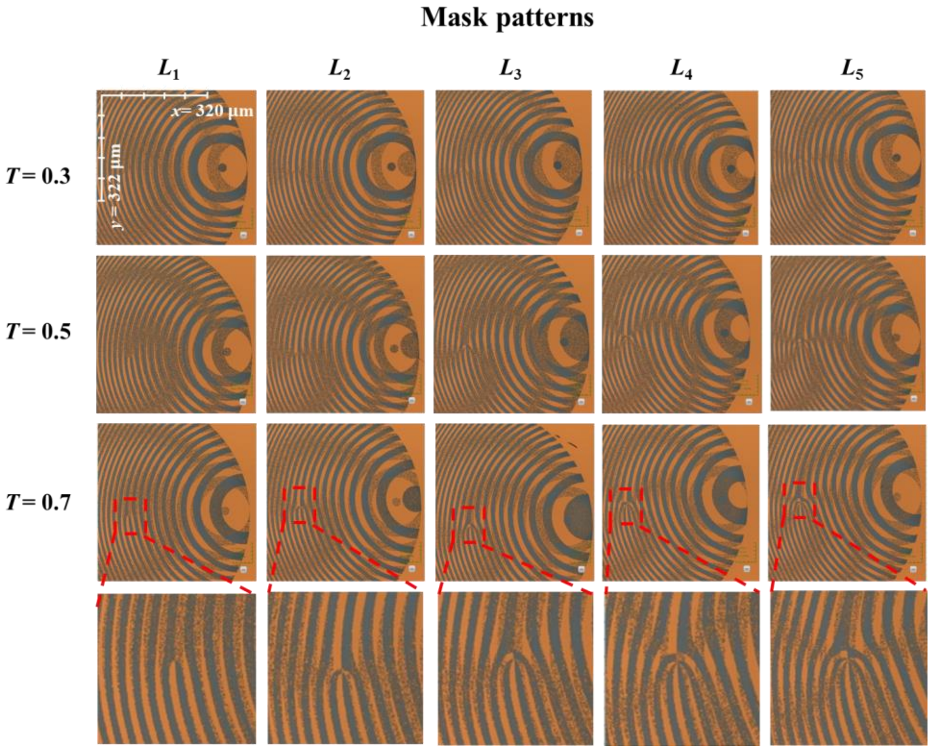

Supplementary Figure S1: Microscope images of the MDOE masks for different topological charges L=1 to 5, splitting ratios T=0.3, 0.5 and 0.7 and a linear phase with an angle α=0.03 fabricated on chromium plates.

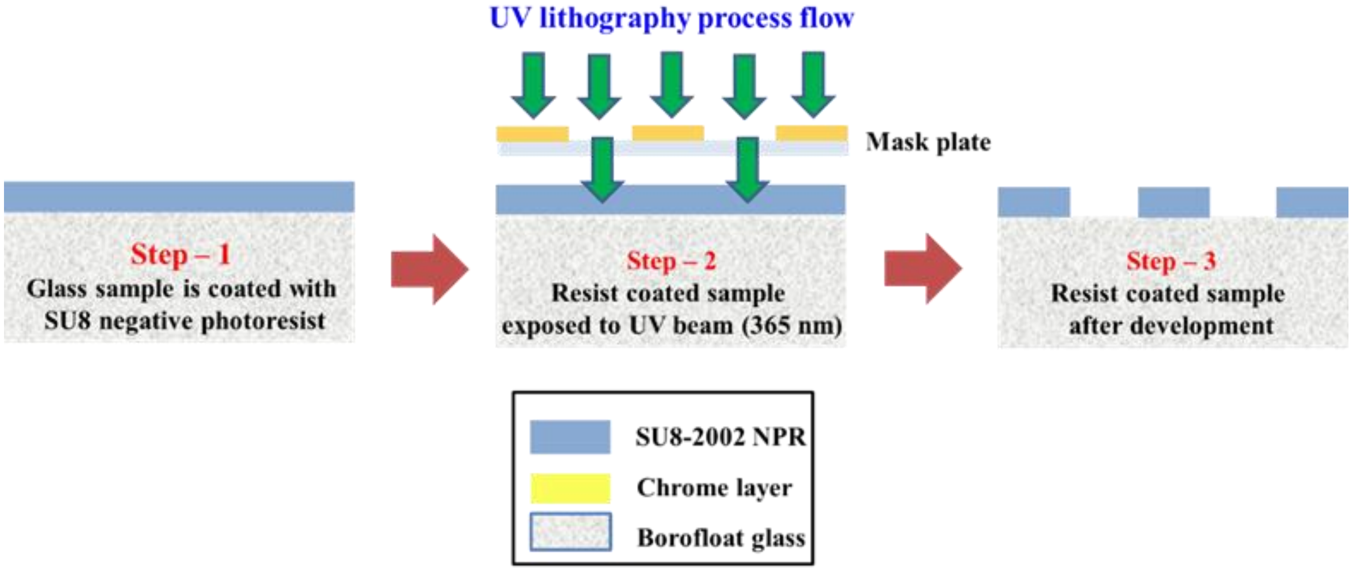

Supplementary Figure S2: Fabrication process for MDOEs using UV lithography.

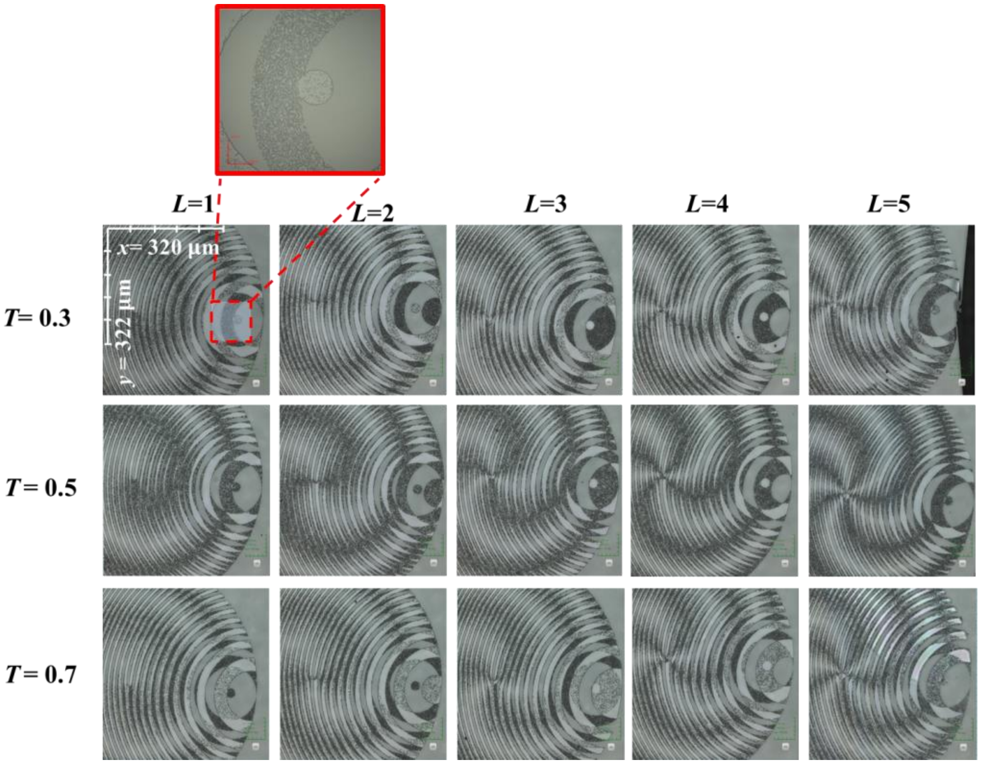

Supplementary Figure S3: Microscope images of the MDOEs for different topological charges L=1 to 5, splitting ratios T = 0.3, 0.5 and 0.7 and a linear phase with an angle α=0.03 fabricated on SU-8 resist layer.

S.2 Simulation results

Simulation was done using Fresnel approximations at λ = 632 nm, other parameters used were a sampling period of 4 μm, and a sampling space of 1000 × 1000 pixels, $f(k_1)$ = 25 cm and $f(k_2)$ = 30 cm. In the first step, the variation in the scattering ratio due to increasing number of layers in the stack of scatterers was studied. A scatterer is designed using Gerchberg Saxton algorithm (GSA) [1-3] with a scattering ratio given by b/B, where B is the length of the spectrum domain and b is the length outside which the intensity is zero. The process is shown in figure S4. A complex amplitude C comprising a constant amplitude (White window at the input) and random phase distribution is Fourier transformed and the resulting amplitude is constrained to only have values within the scattering window of length b while the phase is retained. The process is iterated to obtain the random phase distribution, which will have a scattering ratio of $\sigma = b/B$. The procedure is repeated with different initial random functions and a set of 5 weak scatterers are synthesized with negligible cross-correlation values with b = 20 and B = 1000. The cross-correlation between any two scatterers must be negligible so that when they are stacked, the effective scattering ratio increases. The images of the synthesized independent scatterers and the phase of the stack of weak scatterers when p = 1-5 is shown in figures S5(a)-

S5(e) and figures S5(f)-S5(j) respectively and their respective far-field diffraction patterns are shown in figures S5(k)-S5(o).

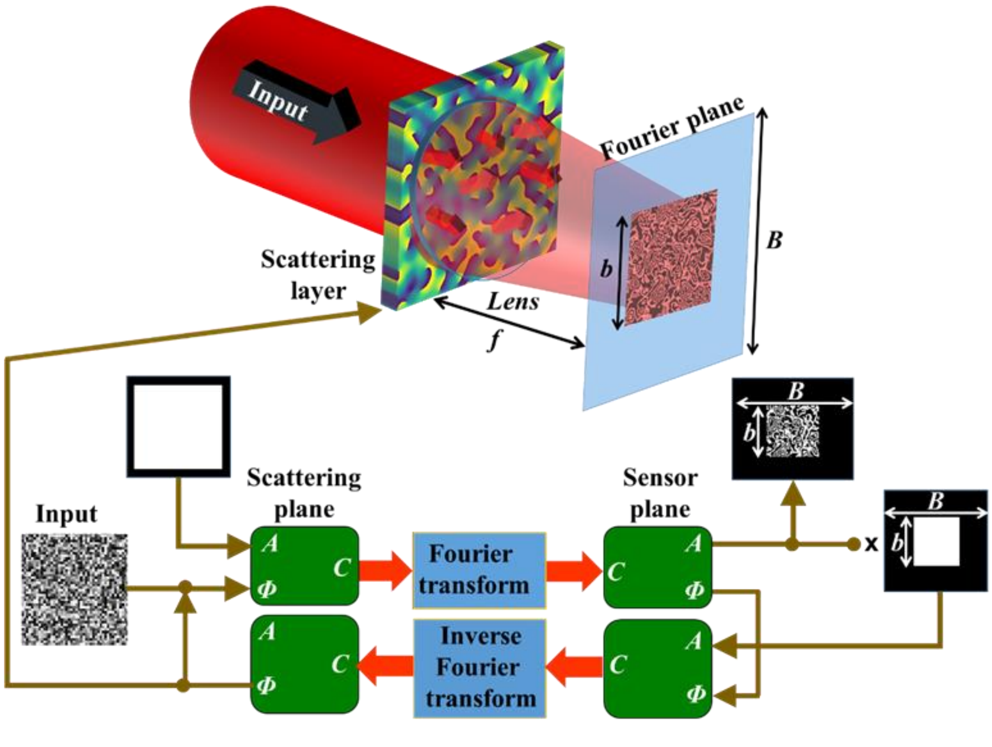

Supplementary Figure S4: Schematic of GSA and design of scattering mask with a scattering ratio of b/B; C- Complex amplitude; A – Amplitude, Φ – Phase, B – Length of the matrix and b – length of the constraint.

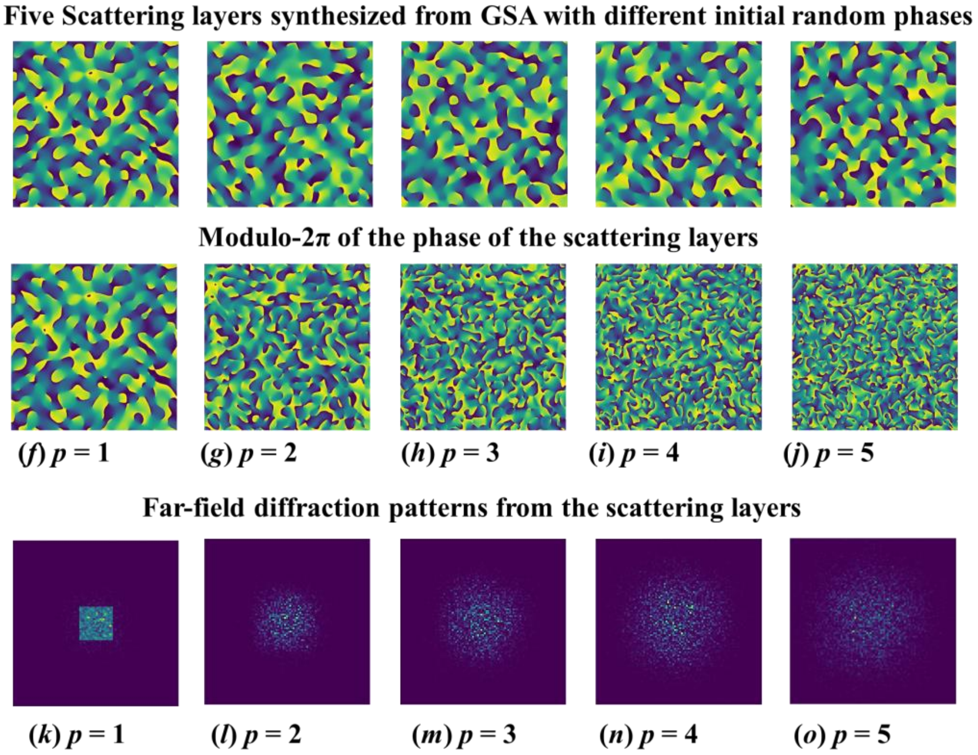

Supplementary Figure S5: Phase images of the scattering layers synthesized from GSA with a scattering ratio of *b*/*B*. Phase images of modulo-2π phase addition of multiple scattering layers for (a) $p = 1$, (b) $p = 2$, (c) $p = 3$, (d) $p = 4$, (e) $p = 5$.

Beam propagation is simulated through the different optical components in the absence of the stack of scatterers and the intensity patterns at $f(k_1)$= 25 *cm* and $f(k_2)$ = 30 *cm* for different topological charges *L*=1-5 are shown in the figure S6. All simulations were done at $T = 0.5$, unless otherwise stated. The interference pattern at $f(k_2)$ = 30 *cm* is stored as a library for implementation in the pattern recognition correlator [4]. Beam propagation is then simulated in the presence of a scatterer (p=1) capable of creating a maximum phase retardation of 0.2π – π in steps of 0.2π and the corresponding interference patterns for different topological charges are shown in figure S7. In this case, the scattering ratio remains constant but the maximum phase retardation is varied.

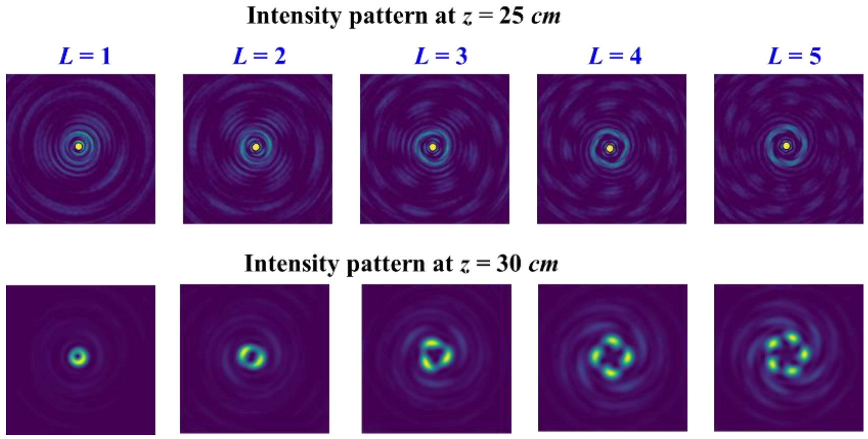

Supplementary Figure S6: Simulated intensity patterns at focal planes $f(k_1)$ =25 *cm* and $f(k_2)$ =30 *cm* for topological charges *L*=1 to 5.

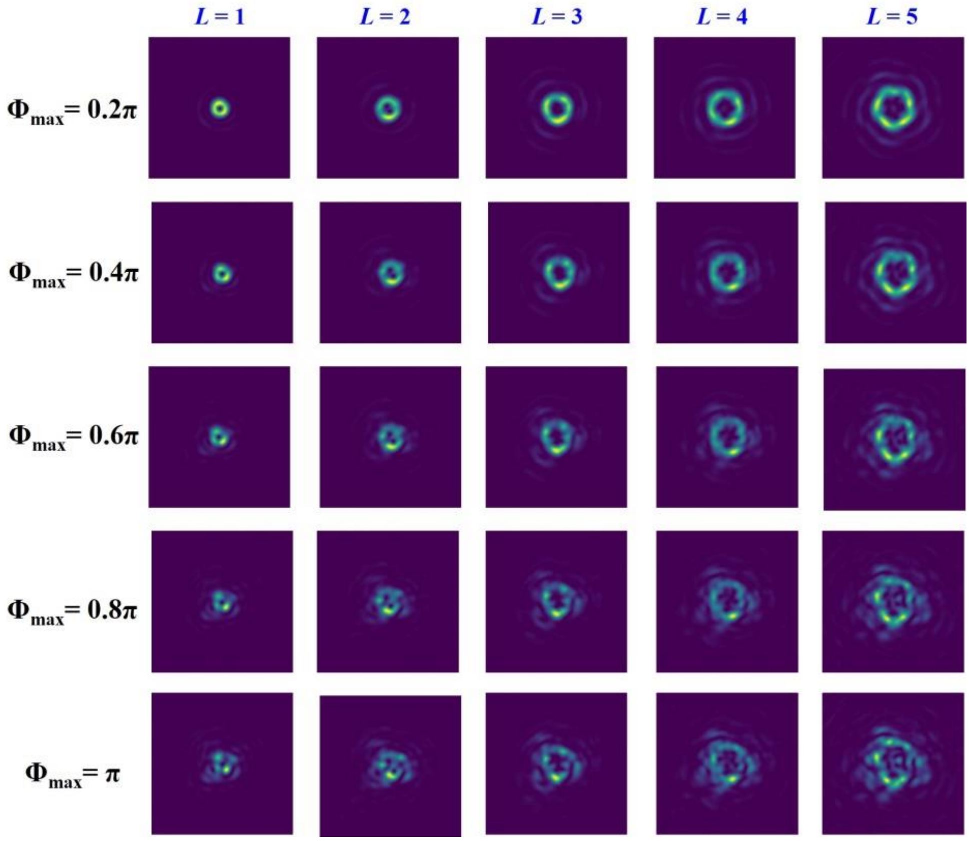

Supplementary Figure S7: Interference patterns for maximum phase retardation of 0.2π-π in steps of 0.2π and topological charges *L*=1 to 5.

The interference patterns for the case for different number of layers p = 1, 2 and 4 but for a maximum phase retardation of 0.6π in each case are shown in rows (1-3) of figure S8. In this case, the scattering ratio is varied but the maximum phase retardation was maintained constant and it is seen that with an increase in the scattering ratio, the results improve contrary to the belief that strong scatterers distort more compared to weaker counterparts. However, when the phase retardation was increased along with the scattering ratio, the behavior reversed. The interference patterns for the case of 0.6π phase retardation for p = 1, 2 and 4 with a maximum phase retardation of 0.6π, 1.2π and 1.8π are shown in figure S9. In this case both the phase retardation as well as the scattering ratio were increased.

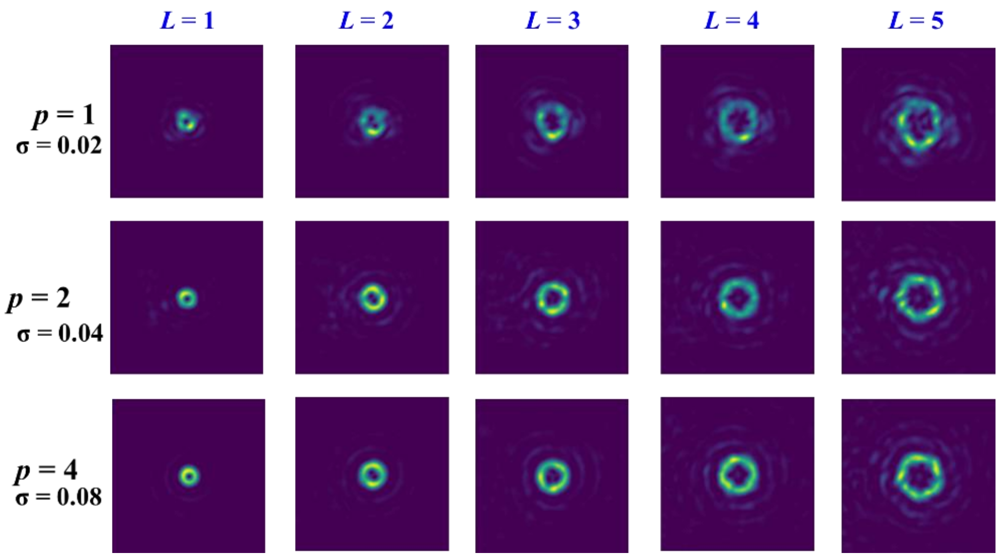

Supplementary Figure S8: Interference patterns for different scattering ratios and topological charges L=1 to 5.

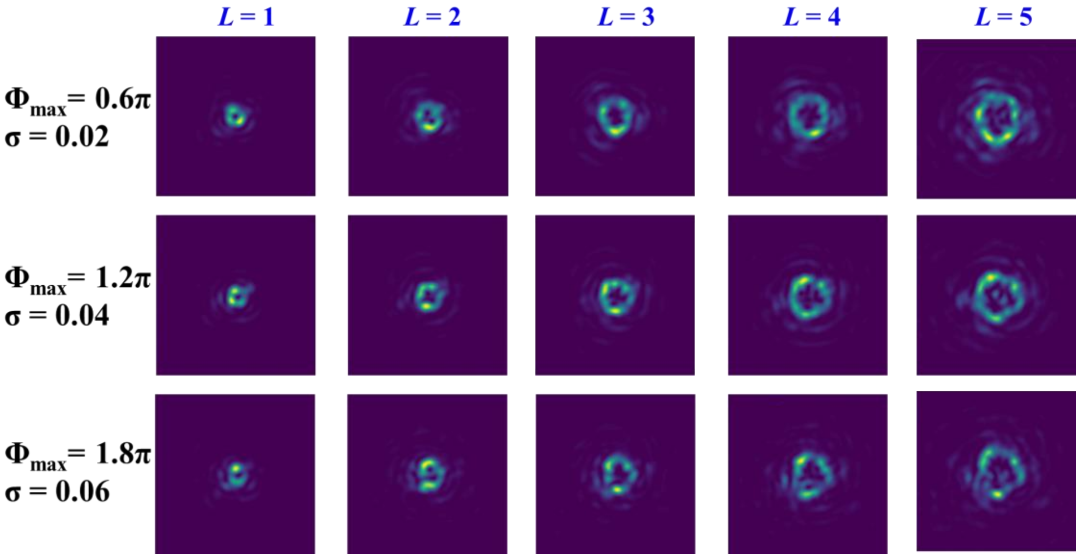

Supplementary Figure S9: Interference patterns for different number of layers and topological charges L=1 to 5.

The cross correlation results for a few of the above cases: L=3 for different phase retardation case with the respective elements from the library in the absence of the scattering layers are shown in figure S10. From the above observations, it can be seen that it is possible to measure the degree of deterioration of the vortex beam through thin scattering layers using a regular matched filter. The narrower the correlation function, better is the match between the two patterns. As seen from the Fig. 5, the broadening matches with the deterioration in the vortex signal. Therefore, the correlation technique can be used as a blind method to quantitatively measure the deterioration of the vortex signal.

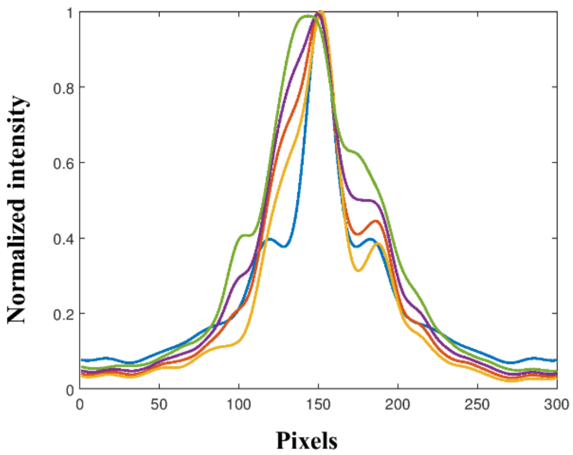

Supplementary Figure S10: Plot of the correlation results for L=3 between different phase retardations (0.2π-π in steps of 0.2π) and the case in the absence of a scatterer. Blue – 0.2π, Yellow – 0.4π, Meroon – 0.6π, Violet – 0.8π and Green – π.

S.3 Study of scattering characteristics of scattering stack

The scattering characteristics of a stack of a scatterer was studied using an experimental setup as shown in figure S11. Light from a He-Ne laser (λ= 632.8 *nm*) is passed through a neutral density filter and a stack of scatterers. The stack of scatterers was created by stacking one scatterer over the other. The maximum scattering degree of the scatterer is measured using trigonometry as θ_{max}= (x/2L). From the scattering degree, the maximum period of the scatterer can be approximated as Λ_{max}=(λ/sin θ_{max}). The values of the scattering degree, period, etc., for the different number of scatterers that make the stack are given in Table – S1. With an increase in the number of layers, the scattering degree increased, while the effective scattering period decreased as described in the previous section.

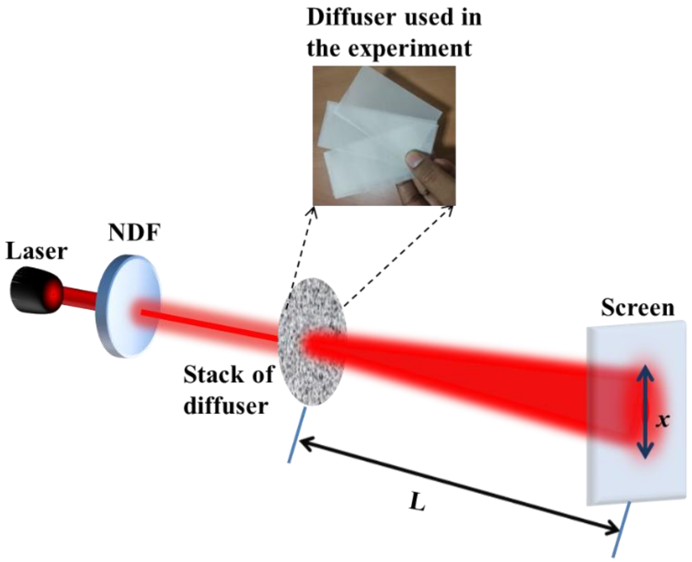

Supplementary Figure S11: Experimental setup for studying the scattering characteristics of a stack of scatterer.

Supplementary Table S1. Scattering parameters for differnet number of scatterers

Number of Scattering layers	x (mm)	L (mm)	θ_{max} (degrees)	Λ_{max} (μm)
1	20	40	14	2.6
2	50	40	32	1.2
3	70	40	41.2	0.96
4	90	40	48.4	0.85

References

1. A. Vijayakumar, Y. Kashter, R. Kelner, and J. Rosen, “Coded aperture correlation holography system with improved performance [Invited],” Appl. Opt. **56**(13), F67–F77 (2017).
2. R. W. Gerchberg, and W. O. Saxton, “A practical algorithm for the determination of phase from image and diffraction plane pictures,” Optik **35**(2), 227–246 (1972).
3. A. Vijayakumar and J. Rosen, "Interferenceless coded aperture correlation holography–a new technique for recording incoherent digital holograms without two-wave interference," Opt. Express **25**(12), 13883-13896 (2017).
4. J. L. Horner and P. D. Gianino, "Phase-only matched filtering," Appl. Opt. **23**(6), 812-816 (1984).
